# Efficacy and safety of radiofrequency ablation for benign thyroid nodules in patients with previous thyroid lobectomy

**DOI:** 10.1186/s12880-021-00577-5

**Published:** 2021-03-11

**Authors:** Lin Yan, Mingbo Zhang, Fang Xie, Jun Ma, Jing Xiao, Yukun Luo

**Affiliations:** Department of Ultrasound, First Medical Center, Chinese PLA General Hospital, No. 28 Fuxing Road, Haidian District, Beijing, China

**Keywords:** Thyroid, Benign thyroid nodule, Radiofrequency ablation, Ultrasound, Volume reduction rate

## Abstract

**Background:**

Radiofrequency ablation (RFA) is recommended for the treatment of benign thyroid nodules. However, data on the clinical role of RFA for benign thyroid nodules in patients with history of thyroid lobectomy are insufficient. The purpose of this study was to evaluate the efficacy and safety of radiofrequency ablation (RFA) for benign thyroid nodules in patients who had previously undergoing thyroid lobectomy.

**Methods:**

From May 2015 to October 2018, a total of 20 patients (19 females, 1 male, mean age 49.50 ± 14.26 years, range 22–74 years) with 20 benign thyroid nodules (mean volume 15.04 ± 21.17 ml, range 0.40–69.67 ml) who had undergone previous thyroid lobectomy were included in this retrospective study. Patients were followed up at 3, 6, 12 months after RFA and every 12 months thereafter by ultrasound, clinical evaluation and thyroid function. Volume, volume reduction rate (VRR), symptom score and cosmetic score were evaluated.

**Results:**

During the mean follow-up time of 21.24 ± 16.41 months, the mean nodule volume decreased significantly from 15.04 ± 21.17 ml to 1.29 ± 1.17 ml (*P* = 0.018) with a mean VRR of 85.41 ± 12.17%. Therapeutic success was achieved in a single session for all thyroid nodules. The symptom score (*P* = 0.001) and cosmetic score (*P* = 0.001) were both significantly reduced at the last follow-up. The levels of free triiodothyronine (fT3), free thyroxine (fT4) and thyroid stimulating hormone were not significantly different at the last follow-up from those prior to treatment (all *P* > 0.05). No life-threatening complications or sequelae occurred after RFA.

**Conclusions:**

As a minimally invasive modality, RFA was a safe, effective, and thyroid function-preserving option for patients with symptomatic benign thyroid nodules after a previous lobectomy.

## Background

Thyroid nodules are common in the general population and occur in 20–70% of individuals [1]. Thyroid lobectomy or nodule resection is the standard treatment according to the size of nodules [2]. However, after the initial surgery, some patients may develop another nodule with symptomatic or cosmetic problems and also require reoperation [3]. The incidence of complications during the reoperation, such as recurrent laryngeal nerve (RLN) injury and hypoparathyroidism is much higher than during the initial surgery because of the distorted anatomy of the thyroid and postoperative adhesions [4–6]. Additionally, patients often need thyroid hormone supplementation after the reoperation, which may have adverse effects on the bones and the cardiovascular system [2]. Therefore, the treatment of benign thyroid nodules in patients with previous thyroid lobectomy often poses a dilemma for both patients and physicians.

Radiofrequency ablation (RFA) and other thermal ablation techniques, such as microwave ablation, laser ablation and high-intensity focused ultrasound (HIFU) ablation have been recommended as safe and effective treatments for benign thyroid nodules by guidelines [2, 7–12]. Several studies have reported a significant reduction in the volume of the nodules along with an improvement in local symptoms or cosmetic problems [13–21]. A meta-analysis reported that the volume reduction rates (VRR) for benign thyroid nodules at 6, 12 and 24 months after RFA were 68%, 75% and 87%, respectively [17]. Moreover, a longitudinal 5-year observational study showed that VRR was 81%, 75%, and 65% for nodules < 10 ml, 10 to 20 ml and > 20 ml, respectively [22], suggesting that the VRR differed depending on the initial volume. However, for patients with benign thyroid nodules after previous lobectomy, only two studies have reported the clinical outcomes of RFA. Ha et al. [23] found that the nodule volume was 9.7 ml in patients who had undergone previous lobectomy before RFA, which decreased significantly at the last follow-up with a mean VRR of 87.2%. Kim et al. [24] reported the benign thyroid goiter developed after unilateral lobectomy decreased significantly from 4.49 ± 0.99 ml to 1.05 ± 0.6 ml with a mean VRR of 81.2 ± 10.5%. Although the volume reduction after RFA was significant in these two studies, the initial volume was small (< 10 ml). Consequently, additional information about the clinical application of RFA for benign thyroid nodules with a larger volume in patients with previous lobectomy is needed.

Therefore, the purpose of this study was to evaluate the efficacy and safety of RFA for benign thyroid nodules in patients with a history of lobectomy.

## Methods

The Institutional Review Board of Chinese PLA General Hospital approved this retrospective study. All the patients were provided written information consent before RFA.

### Patients

Postoperative thyroid nodule was defined as new lesion in the remnant tissue or enlargement in the remaining contralateral lobe after initial surgery [25]. All the enrolled patients fulfilled the following criteria: (1) nodules should be confirmed as benign via two separated fine-needle aspiration (FNA) or core-needle biopsy (CNB); (2) no suspicious malignant features on ultrasound (US) [2, 26]; (3) underwent initial thyroid surgery for benign thyroid nodule; (4) complaint of cosmetic or symptomatic problems or concerns of nodules growing rapidly or malignant transformation; (5) follow-up time ≥ 6 months. The exclusion criteria were: (1) follicular neoplasm or malignancy findings on US-guided FNA or CNB; (2)postoperative nodule with benign result in biopsy but was suspected of malignancy in US; (3) contra-lateral vocal cord paralysis; (4) coagulation disorder or serious heart failure/ respiratory failure/ liver failure/ renal failure; (5) follow-up time < 6 months.

From May 2015 to October 2018, 22 patients with benign nodules who had a history of thyroid lobectomy underwent RFA in this institution. Among them, patients with follow-up time < 6 months (N = 2) were excluded. The remaining 20 patients with 20 benign nodules were included in this study.

### Pre-ablation assessment

All the patients underwent laboratory tests included complete blood count, coagulation tests and thyroid function tests. The thyroid function tests included free triiodothyronine (fT3, normal range 2.76–6.30 pmol/l), free thyroxine (fT4, normal range 10.42–24.32 pmol/l) and thyroid stimulating hormone (TSH, normal range 0.23–5.50 mU/l). Before treatment, each nodule underwent US to assess the size, location, component, margin, shape, echogenicity, calcification and vascularity. The volume of thyroid nodules was calculated with the equation: V = πabc/6 (V is the volume, while a is the largest diameter, b and c are the other two perpendicular diameters) [27]. The nodules were further categorized into three subgroups according to volume as the small (i.e., < 10 ml, N = 12), medium (i.e., 10–30 ml, N = 5), and large (i.e., > 30 ml, N = 3) groups [27]. Before RFA, symptom score was self-measured by patients using a 10-cm visual analogue scale (grade 0–10) [7]. The cosmetic score was assessed by a physician (1, no palpable mass; 2, no cosmetic problem but palpable mass; 3, a cosmetic problem on swallowing only; and 4, a readily detected cosmetic problem) [7].

### RFA procedure

US and contrast-enhanced ultrasound (CEUS) before and after RFA, as well as during follow-up were performed using a Siemens Acuson Sequoia 512 Ultrasound System (Siemens, Mountain View) with a 15L8W linear array transducer or a Philips iU22 Ultrasound System (Philips Healthcare) with a L12-5 linear array transducer or a Mindray M9 Ultrasound System (Mindray) with a L12-4 linear array transducer. US-guided FNA or CNB and RFA were all performed using a Siemens Acuson Sequoia 512 Ultrasound System with a 6L3 linear array transducer.

**Fig. 1:**
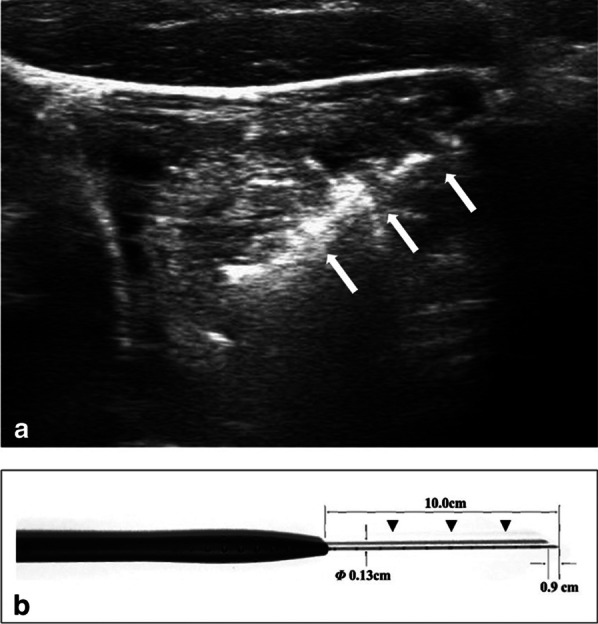
The 18-gauge bipolar RFA needle used in this study. The ultrasound image of the 18-gauge bipolar RFA needle (arrows) during the RFA procedure for benign thyroid nodules (**a**). The needle had insulated metallic sheath (arrowheads) and the active tip was 0.9 cm (**b**)

CEUS was performed to assess the blood supply of the nodule before and immediately after ablation. The US contrast agent was sulphur hexafluoride (SonoVueR, 2.4 ml) followed by a 5 ml normal saline flush. All RFA procedures were performed by an experienced US physician with more than 20-year experience in interventional thyroid US. A bipolar RFA generator (CelonLabPOWER, Olympus Surgical Technologies Europe) and an 18–gauge bipolar RF electrodes with 0.9 cm active tip were used (CelonProSurge micro 100-T09, Olympus Surgical Technologies Europe) in this study (Fig. 1).

Patients were placed an operating table in the supine position with neck extended. Local anesthesia with 1% lidocaine was administered. The hydrodissection technique was usually used if the distance between the tumor and critical cervical structures (trachea, cervical artery, jugular vein, esophagus and recurrent laryngeal nerve) was < 5 mm [7]. Considering the nodular volume, distorted anatomy and postoperative adhesions caused by the previous surgery, this technique was performed to all the patients in this study. The RFA power was 3 W. If a transient hyperechoic zone did not form at the electrode tip within 5–10 s, the radiofrequency power was increased to 5–9 W [28]. CEUS was performed immediately after the RFA procedure to evaluate the ablation area. If any enhancement existed, a complementary ablation could be performed [28]. During the procedure, special attention was given to the protection of critical cervical structures in order to prevent thermal injury or complication. Each patient was observed for 2 h in the hospital while any complication occurring during and immediately after ablation were carefully evaluated according to the clinical signs and symptoms [28].

After RFA, patients were followed up at 3, 6, 12 months and every 12 months thereafter and underwent periodic US, clinical evaluation and thyroid function. Symptom scores, cosmetic scores and complications after RFA were evaluated at each follow-up. The volume reduction was calculated as follows: VRR = ([initial volume-final volume] × 100%)/initial volume [27]. Therapeutic efficacy was defined as a > 50% volume reduction at last follow-up [27]. Regrowth was defined as the nodule volume increased > 50% compared to the previously recorded volume [27, 29]. Additional ablation may be considered if the nodule showed marginal regrowth or if cosmetic or symptomatic problems were incompletely resolved [7].

### Statistical analysis

Statistical analysis was performed using the SPSS statistical software (Version 25.0). Continuous data were expressed as mean ± SD (range). Wilcoxon signed rank tests were used to compare the mean volume, symptom and cosmetic scores before RFA and at each follow-up point after RFA. A difference with *P* < 0.05 was considered as statistically significant.

## Results

### Patient characteristics

Clinical characteristics of patients before RFA are presented in Table 1. A total of 20 patients (19 females, 1 male) with 20 benign thyroid nodules who had a history of thyroid lobectomy were included in this study. The mean age was 49.50 ± 14.26 years. The mean initial volume was 15.04 ± 21.17 ml.

**Table 1:** Clinical characteristics of patients before RFA

Characteristics	Data
No. of patients	20
No. of nodules	20
Age (years)	49.50 ± 14.26
Sex (F/M)	19/1 (95.00/5.00)
largest diameter (cm)	3.18 ± 1.46
Mean volume (ml)	15.04 ± 21.17
Small (N = 12)	4.24 ± 3.63
Medium (N = 5)	12.90 ± 1.66
Large (N = 3)	61.83 ± 13.56
Location	
Right lobe	10 (50.00)
Left lobe	9 (45.00)
Isthmus	1 (5.00)
Hypothyroidism before treatment	1 (5.00)

Values are presented as mean ± SD (range) or number of tumors (percentages)

*RFA* radiofrequency ablation

In RFA procedure, power of 3 W was used in 2 nodules; 5–6 W was used in 7 nodules; 7–8 W was used in 7 nodules and 9 W was used in 4 nodules. The mean RFA time was 365 ± 182.50 s and the mean energy was 2337.89 ± 1379.74 J. The mean energy applied per volume was 628.79 ± 861.45 J/ml.

### Efficacy

The volume and VRR at each follow-up point after RFA are summarized in Table 2. The mean volume decreased significantly from 15.04 ± 21.17 ml to 1.29 ± 1.17 ml. The VRR was 62.81 ± 21.56%, 74.83 ± 13.44%, 81.65 ± 13.59% and 85.42 ± 12.16% at 3, 6, 12 and 24 months, respectively. All the nodules underwent a single session, and the therapeutic efficacy rate was 100%. The VRR was 86.71 ± 14.11%, 82.19 ± 7.95%, and 75.65 ± 3.73% in the small, medium, and large group, respectively (Table 3). No regrowth was found after RFA. Two nodules (10.00%) in the small group were disappeared during the follow-up. At the last follow-up, the symptom scores significantly decreased from 3.00 ± 2.64 to 0.90 ± 1.33 (*P* = 0.001), and the cosmetic scores significantly decreased from 2.40 ± 1.23 to 1.40 ± 0.60 (*P* = 0.001).

**Table 2:** Changes of volume and VRR at each follow-up after RFA

Time (months)	Volume (ml)	*P* value (vs initial volume)	VRR (%)
3	11.53 ± 16.88	0.005	62.81 ± 21.56
6	6.24 ± 8.74	0.002	74.83 ± 13.44
12	3.94 ± 5.70	0.003	81.65 ± 13.59
24	1.29 ± 1.17	0.018	85.42 ± 12.16

RFA: radiofrequency ablation

**Table 3:** Changes of VRR in subgroups at each follow-up after RFA

Time (months)	Small group (N = 12)	Medium group (N = 5)	Large group (N = 3)
3	73.73 ± 25.93	60.82 ± 9.57	50.22 ± 22.58
6	79.01 ± 16.96	72.35 ± 11.59	68.95 ± 5.20
12	85.29 ± 16.22	74.93 ± 0.82	75.65 ± 3.73
24	86.71 ± 14.11	82.19 ± 7.95	NA

*NA*: not available

*VRR*: reduction volume rate, *RFA* radiofrequency ablation

**Fig. 2:**
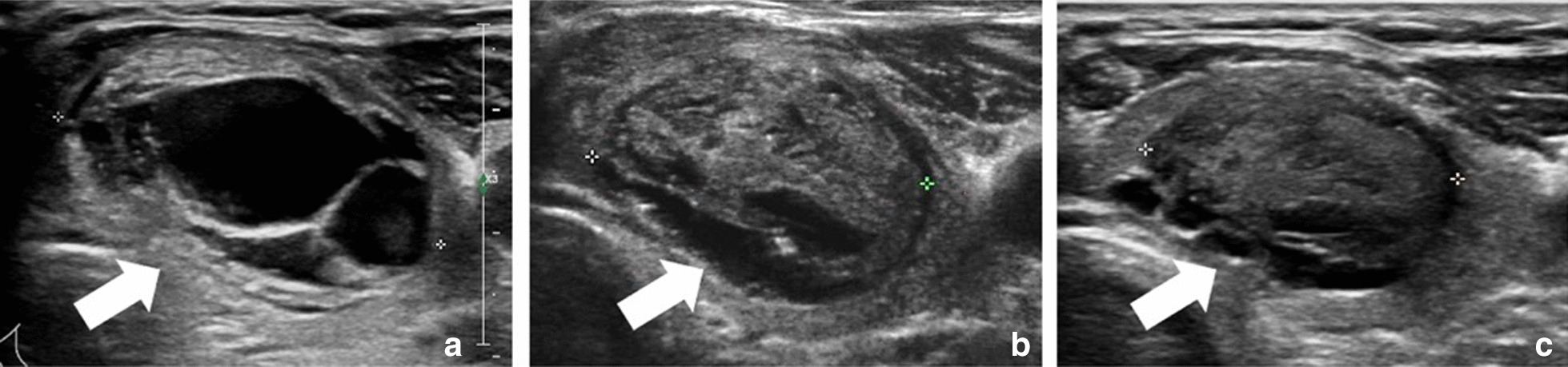
US images of a postoperative thyroid nodule. **a** The transverse US showed a postoperative nodule at the left lobe. Before RFA, the initial volume was 11.95 ml. **b** At 3 months after RFA, the volume of nodule was 5.43 ml. C. At 12 months after RFA, the volume shrunk to 2.92 ml

The changes of fT3, fT4 and TSH are before RFA and at last follow-up were summarized in Table 4. The thyroid function was well-maintained after RFA and no patient developed hypothyroidism. A representative case before and after RFA is shown in Fig. 2.

**Table 4:** The changes of fT3, fT4 and TSH before RFA and at last follow-up

	Baseline	At last follow-up	*P* value
fT3	4.98 ± 0.87	4.67 ± 0.51	0.180
fT4	16.41 ± 4.41	15.24 ± 2.12	0.655
TSH	1.22 ± 0.69	1.67 ± 0.74	0.180

*fT3* free triiodothyronine, normal range 2.76–6.30 pmol/l, *fT4* free thyroxine, normal range 10.42–24.32 pmol/l, *TSH* thyroid stimulating hormone, normal range 0.23–5.50 mU/l, *RFA* radiofrequency ablation

### Safety

All the patients tolerated the RFA procedure. No patients had complications during or after RFA. Side effects like pain and discomfort occurred in 8 patients, which resolved spontaneously within 3 days.

## Discussion

This study showed that during a mean follow-up time of 21.24 ± 16.41 months, the mean VRR of benign nodules in patients with previous lobectomy was 85.41 ± 12.17%, while the therapeutic efficacy rate was 100%. All nodule-related symptoms and cosmetic problems showed clinical improvement without the occurrence of any life-threatening complications or sequelae after RFA. Moreover, thyroid function was well-maintained after RFA, and no patient developed hypothyroidism. These results demonstrated that RFA was a safe, effective and thyroid function preserving treatment for patients with previous lobectomy, even for large thyroid nodules (> 10 ml).

Although RFA and other thermal ablation techniques has been considered as effective treatments for benign thyroid nodule [2, 7–12], evidence regarding the clinical outcomes of ablation for nodules in patients with previous lobectomy was limited. Ha et al. [23] first reported that the nodule volume was 9.7 ml in patients who had undergone lobectomy before RFA, and it was significantly decreased at the last follow-up with a mean VRR of 87.2%. Kim et al. [24] found that RFA resulted in a mean VRR of 81.2 ± 10.5% in patients with a benign thyroid goiter who had a history of unilateral lobectomy. A similar VRR was also observed in this study (85.41 ± 12.17%) after a mean follow-up time of 21.24 ± 16.41 months. Compared with previous studies that only included small volume nodules (< 10 ml) [23, 24], the initial volume in this study was much larger (15.04 ± 21.17 ml). Moreover, the therapeutic efficacy rate in every group was 100%, and all nodule-related symptoms and cosmetic problems showed clinical improvement. These results indicated that RFA of larger (> 10 ml) nodules in patients with previous lobectomy was also effective.

Although reoperation is the standard treatment for postoperative symptomatic benign nodules, it is associated with a high incidence of complications because of the normal tissue plane distortion and scar formation due to the initial surgery [4, 5, 30, 31]. RLN injury and hypoparathyroidism are the major complications. Hardman et al. [31] reported that after reoperation, the incidence of transient RLN injury was 0–22% and of permanent RLN injury was 0–13% [31]. The incidence rates of transient and permanent hypoparathyroidism were 56.6% and 10%, respectively [4]. However, in this study, no life-threatening complications or sequelae were observed after RFA. This was consistent with the findings of a recent meta-analysis that showed the complication rate after RFA was only 1.44% [32]. There were several reasons for the low incidence of complications after RFA. First, during the RFA procedure, real-time US imaging allowed the physician to ablate the target nodule accurately while carefully monitoring the critical structures [33]. Second, the RFA procedure was performed by an experienced US physician. The experience of the physician was found to be an important factor for preventing thermal injury to the critical structures or nerves [7], particularly in patients with a distorted anatomy and postoperative adhesions from the initial surgery. Third, the moving-shot technique and hydrodissection technique were used during the RFA procedure, which have been verified as safe methods for ablating the nodule margin and preventing thermal injury [7].

Due to the different types of the initial thyroid surgery as well as different sizes of the postoperative nodules, reoperation may involve partial or total thyroidectomy. Patients often need life-long thyroid hormone supplementation, which leads to adverse effects on the bones and the cardiovascular systems [1, 2, 34]. Since only the targeted nodule was ablated via real-time US monitoring, the incidence of permanent hypothyroidism after RFA was only 0.04% (1/2245) [32]. Ha et al. [23] reported that RFA did not affect thyroid function in patients with postoperative benign thyroid nodules. In the present study, the thyroid function of the patients was well maintained, and no patient developed hypothyroidism after RFA. Hypothyroidism after RFA was rare, and the main cause of hypothyroidism seems to be a progression of autoimmune thyroiditis associated with preexisting thyroid antibodies [23]. Although the probability of immunological activation and subsequent hypothyroidism after ablation was low, patients with thyroid antibodies before treatment should be informed the possibility of hypothyroidism.

In this study, bipolar RFA was used for treatment, because it had more advantages than monopolar RFA, especially when used in sensitive cervical areas [35–38]. Monopolar RFA required a grounding pad so that the electrical current could flow between the electrode and the grounding pad, which could lead to painful skin burns, interfer with implanted cardiac devices, and could limit the efficiency when placed incorrectly [35–38]. In contrast, in bipolar systems, the electric current was limited to the applicator tip, which could eliminate the side effects of monopolar RFA [36], resulting in a lower risk complication [8]. Moreover, monopolar RFA lost more energy to the tissue within the electrical circuit because of the higher distance to the grounding pole; thus it was more likely to stimulate sensory nerve structures and consequentially cause pain [36].

There were some limitations of this study. Firstly, it was a single-center retrospective study. Secondly, the sample size was small, and the follow-up time was relatively short. Thirdly, benign confirmation of the postoperative thyroid nodules was carried out via two separate FNAs or CNBs. False negative results or the presence of occult microcarcinoma could not be completely excluded. Lastly, because the thyroid function before RFA was normal in all patients, thyroid antibody tests were not performed after RFA.

## Conclusions

As a minimally invasive modality, RFA was a safe, effective and thyroid function- preserving option for patients with symptomatic benign thyroid nodule after a previous lobectomy.

## Data Availability

The datasets generated and/or analyzed during the current study are not publicly available due patient privacy but are available from the corresponding author on reasonable request.

## Abbreviations

RFA: Radiofrequency ablation
VRR: Volume reduction rate
FNA: Fine-needle aspiration
CNB: Core-needle biopsy
RLN: Recurrent laryngeal nerve
